# Working Towards Holistic Scar Assessment and Improved Shared Decision Making in Global Burn Care

**DOI:** 10.1093/jbcr/irad089

**Published:** 2023-06-13

**Authors:** Milly S van de Warenburg, Elleke F L Munk, Anna Davies, Craig A McBride, Dale W Edgar, Mariëlle L A W Vehmeijer-Heeman, Amber E Young

**Affiliations:** Department of Plastic and Reconstructive Surgery, Radboud University Medical Centre, Radboud Institute for Health Sciences, Nijmegen, The Netherlands; Department of Plastic and Reconstructive Surgery, Radboud University Medical Centre, Radboud Institute for Health Sciences, Nijmegen, The Netherlands; Translational Health Sciences, University of Bristol, Bristol, UK; Centre for Children’s Burns and Trauma Research, Child Health Research Centre, The University of Queensland, Brisbane, QLD, Australia; State Adult Burn Unit, Fiona Stanley Hospital, Murdoch, Australia; Burn Injury Research Node, The Institute for Health Research, The University of Notre Dame Australia, Fremantle, Australia; Fiona Wood Foundation, Fiona Stanley Hospital, Murdoch, Australia; Department of Plastic and Reconstructive Surgery, Radboud University Medical Centre, Radboud Institute for Health Sciences, Nijmegen, The Netherlands; Centre for Surgical Research, Population Health Sciences, Bristol Medical School, Bristol University, Bristol, UK

## Abstract

Cutaneous burn scars impact various aspects of life. Scar treatment is mainly evaluated on scar characteristics. Consensus is needed on which other outcomes to capture, ensuring they are relevant to patients, clinicians, and researchers. The aim of this study was to identify, discuss and analyze outcomes related to cutaneous burn scarring, incorporating the voice of patients and views of healthcare professionals. For this, a Delphi process consisting of two survey rounds and a consensus meeting was initiated. Burn scar-related outcomes were identified from an existing comprehensive list of 100 outcomes by an international panel of patients, healthcare professionals and researchers. Fifty-nine outcomes were identified from the Delphi process as related to scarring (≥60% votes). Outcomes less impactful in relation to scar outcomes included psychosocial issues, sense of normality, understanding of treatment, costs and systemic issues. To represent a holistic assessment of outcomes related to cutaneous burn scarring, this Delphi process established a battery of outcomes currently included in scar quality assessment tools, and an expanded set of less frequently considered outcomes. Future work in this area must include the patient voice from developing countries. This is essential to identify globally applicable outcomes related to scarring.

Burn patients cannot be considered recovered when their wounds have re-epithelialized.^1^ With a declining mortality rate after a burn injury since 1990, more patients must live with the challenges of post-burn sequelae, such as cutaneous scars.^2^ Pathological scarring commonly occurs after a burn, manifesting itself as scar hypertrophy, limited range of motion, contractures, and/or keloids.^3,4^ Scar management options include pressure garments, silicone, laser therapy, corticosteroid injections, and/or reconstructive surgery.^5,6^ Treatment efficacy is monitored using scales such as the Vancouver Scar Scale (VSS)^7^ or Patient and Observer Scar Assessment Scale (POSAS),^8^ evaluating scar characteristics such as pain, itch, thickness, pliability, and color.^9^

While the emphasis of burn scar treatment focuses on improving scar quality, burn scars can cause long-term (LT) physical and psychosocial morbidity, negatively impacting patients’ health-related quality of life.^3,10^ Scar-related challenges during recovery include psychological distress, overcoming functional issues and facing difficulties in daily life impacting work, sports, relationships, and school.^11,12^ Several patient-reported outcome measures (PROMs) are gathering broader application, enabling assessment of these nonphysical impacts of a burn scar.^13–15^

Since burn scarring has a multi-faceted impact and requires holistic assessment for adequate individualization of treatment.^11^ A recent systematic review on qualitative research showed a diversity of outcomes associated with burn scarring, some of which are not currently assessed in scar assessment scales or other available PROMs.^16^ Clinical outcomes of potential importance have not yet been evaluated. The aim of this study is to explore the diversity of cutaneous burn scar-related outcomes captured in the clinical and research setting and reach consensus on those that are considered to be related to cutaneous scarring using a previously developed comprehensive list of burn outcomes.

## METHODS

### Study Design

This study is a secondary analysis of data from the Core Outcome Set in Burn Care Research international (COSB-i), which was published recently.^17^ A Core Outcome Set (COS) is an agreed minimum set of the most important outcomes that should be reported in all trials of a specific condition.^18^ The COSB-i was a sub-set battery developed from a comprehensive longer list of 100 burn outcomes covering both the acute phase and post-acute phase after burn injury. This list was assembled by identifying relevant outcomes through systematic reviews of clinical^19^ and patient-reported^13,20^ outcomes in burn care, triangulated with semi-structured interviews with patients (adults and young people), caregivers (parents of young people with a burn injury) and healthcare professionals. Next, this initial list of outcomes was refined in a two-round Delphi survey to prioritize and achieve consensus on outcomes of highest importance according to the contributors. Involved panelists included patients and caregivers (n = 126) and health care professionals and burn care researchers (n = 668). The extended methodology for the COSB-i study has been published elsewhere.^21^

For this current study, a Delphi method was applied to reach consensus on which outcomes from the COSB-i long list were burn scar related. All 100 outcomes concerning both the acute and post-acute phase were taken up for consideration. Using the Delphi method, both healthcare professionals and patients can be involved and their feedback can be implemented during the process to reach consensus.^22^ This iterative process consisted of two phases:

1. Phase 1: a survey was undertaken involving two questionnaire rounds using the 100 items identified for the long list in the COSB-i study, to select outcomes considered related to cutaneous burn scarring by professionals and patients.
2. Phase 2: an online meeting was held to achieve consensus on those outcomes that remained undecided after the survey.

### Delphi Panel

There is no agreed standard panel size for Delphi studies.^22^ It is however recommended to state criteria for selection and information on recruitment of the panel.^23^ Panel diversity was pursued in this study to include divergent perspectives, thus enabling innovative and creative discussions.^22^ Healthcare and research professionals were approached to participate based on their experience in scar management and were purposefully selected by the project steering group to represent: 1) a range of roles involved in burn care; 2) involvement with both adult and pediatric patients; and 3) a range of countries. Patient panelists were recruited as public-patient involvement participants through local clinical networks, charities, patient support groups, and word of mouth referrals.

### Data Collection

#### Survey Rounds

There is no agreed amount of survey rounds for Delphi studies.^23^ A two-round structured Delphi survey was chosen since we expected most changes in panelists’ responses in the first two rounds.^24^ No other outcomes could be added in between the survey rounds, since we specifically wanted to identify outcomes from the COSB-i long list. The survey consisted of demographic questions (occupation, experience in burn care in years and country of origin) and the list of outcomes, presented in Microsoft Excel (Microsoft Corporation, 2018). Participants were presented with the full explanation for each outcome as it was presented in the COSB-i survey (Supplementary Table S1). A distinction was made between outcomes that applied to both the acute phase and the LT) such as weight loss or anxiety about the future.

Healthcare professional and patient or caregiver representative panelists were invited to participate by e-mail for the Delphi survey. Information regarding the purpose, its voluntary basis and instructions for the survey were included in the e-mail. Participants were given periods of 6 and 3 weeks for round 1 and 2, respectively to decide whether they wanted to participate and to reply. Reminders were send out every 1–2 weeks to improve the response rate.

- In round 1, each participant was asked to decide for each of the 100 burn outcomes listed if that outcome was directly or indirectly related to scarring using “Yes,” “No,” or “Maybe.”
- In round 2, the participants were presented with anonymized results from the first round by displaying the number of votes on “Yes,” “No,” or “Maybe” for each outcome. Patient/caregiver votes were separately displayed. Each participant was asked to vote again whether the outcome was directly or indirectly related to cutaneous scarring, this time by voting “Yes” or “No.”

Consensus was reached when agreement of at least 80% was achieved in the second round. Outcomes that received either ≥80% “yes” or “no” votes were considered related or unrelated to scarring, respectively. Patients’ votes were weighted, meaning that a majority vote could only be accepted if the patient panelists also unanimously voted along with the majority. A cutoff value of 80% was chosen for the survey rounds to pre-select outcomes that had the most agreement. Undecided votes (<80% concordance) were taken to the consensus meeting for further discussion and final decision making.

#### Consensus Meeting

An international video conference (Zoom version 5.10.4, Zoom Video Communications, Inc.) was held 6 weeks after completing the survey to achieve consensus on the remaining undecided outcomes from the two survey rounds. These outcomes were grouped in categories to facilitate voting: psycho(social), burn wound related, complications, dysphagia/dysphonia, short-term systemic effects, organ dysfunction or failure, pain and discomfort, LT (systemic) effects, hospital stay, treatment, and costs. There was no predetermined maximum number of outcomes that could be selected for the final list of burn scar-related outcomes.

Those who participated in the survey were invited to participate in the consensus meeting. Additionally, international health care professional and UK patient panelists were invited to the meeting via professional burn organizations, burn charities, or through patient support groups. Additional professional panelists were invited from developing countries. Additional UK patient panelists were invited to reduce professional bias and were separately informed prior to the meeting of its purpose via e-mail. Bias was reduced through the use of an independent chair who was not involved in the project and had expertise in Delphi methodology.

Prior to discussion about the relation of each undecided outcome to burn scarring, the purpose of the meeting was discussed with all participants. Next, undecided outcomes which received less than 80% of the votes during the survey were presented, discussed, and voted on. Participants voted anonymously and independently using online polling via Zoom. Participants were able to choose from the options “Relates to scarring,” “Does not relate to scarring” or “Don’t know/no opinion.”

Overall, outcomes that ≥60% of the participants voted as related to cutaneous scarring were considered to be relevant.

## RESULTS

### Delphi Survey Panel Participation

Seventeen participants from four countries agreed to take part in both the first and second survey round (Table 1). Of these, four doctors, six allied health professionals, three nurses, two burn researchers, one patient who sustained a burn injury as a child, and one parent of a child who sustained a burn injury participated. The majority of the health care professionals (13/15) had more than 5 years of experience in burn care.

**Table 1.** Demographics of healthcare professionals and patient stakeholders in the Delphi survey

Demographics	Participants (*n* = 17)
Gender	
Male	5
Female	12
Occupation	
Doctor ([Pediatric] Burn Surgeon, Anesthetist, and Junior Doctor)	4
Allied Health Professional (Physiotherapist, Occupational Therapist, and Psychologist)	6
Burn Care Nurse/Research Nurse	3
Researcher	2
Burns charity	0
Patient	1
Parent	1
Experience in Burn Care	
0–3 yr	2
4–5 yr	0
>5 yr	13
Country of Origin	
Experts	
Australia	5
Belgium	2
The Netherlands	2
United Kingdom	6
Patients/Parents of Patients	
United Kingdom	2

#### Delphi Survey Results

The results of the two survey rounds are displayed in Table 2. Votes from the patient and parent participants are displayed separately in brackets behind the total amount of votes. In the second round, 40 outcomes reached ≥80% agreement and were considered related to scarring (highlighted in Table 2 in green). Ten outcomes in the second round were likewise considered unrelated to scarring, highlighted in red. The remaining 50 outcomes were carried through to the consensus meeting for further discussion and voting.

**Table 2.** Responses from survey round 1 and 2

Outcome	Round 1		Round 2		Outcome	Round 1		Round 2	
Related to Scarring?	Yes/Maybe	No	Yes	No	Related to Scarring?	Yes/Maybe	No	Yes	No
Patient Psychology	17 (2)^*^	0	17 (2)^+^	0	Length of Stay	10 (1)	7 (1)	13 (2)	4
Scar Elasticity	17 (2)	0	17 (2)^+^	0	Inflammatory Markers	9 (1)	8 (1)	13 (1)	4 (1)
Time to Healing	13 (1)	4 (1)	17 (2)^+^	0	Number of Appointments	11 (2)	6	13 (2)	4
Number of Surgeries	15 (2)	2	17 (2)^+^	0	Dignity	9 (1)	8 (1)	12 (2)	5
Adherence to Treatment	16 (2)	1	17 (2)^+^	0	Pain All the Time	12 (1)	5 (1)	12	5 (2)
Anxiety About the Future	17 (2)	0	17 (2)^+^	0	LT Healthcare Costs	11 (2)	6	12 (1)	5 (1)
Understanding of Treatment	14 (2)	3	17 (2)^+^	0	Substance Abuse	11 (1)	6 (1)	11 (1)	6 (1)
LT** Walking	17 (2)	0	17 (2)^+^	0	Dressing Comfort	9 (2)	8	11 (2)	6
LT Scar Contractures	17 (2)	0	17 (2)^+^	0	LT Metabolism	11 (1)	6 (1)	11 (1)	6 (1)
LT Burn Color	17 (2)	0	17 (2)^+^	0	LT Body Weight	11 (1)	6 (1)	11	6 (2)
LT Daily Tasks	17 (2)	0	17 (2)^+^	0	Unplanned Readmission	11 (1)	6 (1)	10 (1)	7 (1)
LT Adherence to Treatment	16 (2)	1	17 (2)^+^	0	Moderate Complications	9	8 (2)	10 (1)	7 (1)
LT Anxiety (About the Future)	17 (2)	0	17 (2)^+^	0	Serious Complications	9	8 (2)	10 (1)	7 (1)
LT Appearance	17 (2)	0	17 (2)^+^	0	Metabolism	10	7 (2)	10 (1)	7 (1)
LT Unwanted Attention	17 (2)	0	17 (2)^+^	0	Suicide	10 (2)	7	9 (2)	8
LT Understanding of Treatment	15 (2)	2	17 (2)^+^	0	Pain During Treatment	8 (1)	9 (1)	9 (1)	8 (1)
LT Use of Creams and Dressings	17 (2)	0	17 (2)^+^	0	Body Weight	9 (1)	8 (1)	8 (1)	9 (1)
LT Number of Surgeries Needed	16 (2)	1	17 (2)^+^	0	Sepsis	7	10 (2)	7 (1)	10 (1)
LT Number of Reconstructions Needed	16 (2)	1	17 (2)^+^	0	Multiorgan Dysfunction	9 (1)	8 (1)	7 (1)	10 (1)
LT Dignity	14 (2)	3	17 (2)^+^	0	Healthcare Costs	6 (1)	11 (1)	7	10 (2)
LT Hair Loss	17 (2)	0	17 (2)^+^	0	Pain in the Donor Site	9 (1)	8 (1)	7 (1)	10 (1)
LT Scar Pain	17 (2)	0	17 (2)^+^	0	Fatigue	9	8 (2)	6	11 (2)
LT Scar Texture	17 (2)	0	17 (2)^+^	0	Multiorgan Failure	8 (1)	9 (1)	6 (1)	11 (1)
LT Itch	17 (2)	0	17 (2)^+^	0	Length of ICU Stay	9 (1)	8 (1)	6 (1)	11 (1)
LT Anxiety (About Treatment)	17 (2)	0	17 (2)^+^	0	Amount of Medication	8	9 (2)	6 (1)	11 (1)
LT Patient Costs	15 (2)	2	17 (2)^+^	0	LT Cognitive Function	7	10 (2)	6	11 (2)
LT Personal Relationships	17 (2)	0	17 (2)^+^	0	Dysphagia	9 (1)	8 (1)	5 (1)	12 (1)
LT Return to Work and School	17 (2)	0	17 (2)^+^	0	Dysphonia	8 (1)	9 (1)	5 (1)	12 (1)
Impact on Family	14 (2)	3	16 (2)^+^	1	Other Infection	9	8 (2)	5 (1)	12 (1)
Breast Development	16 (2)	1	16 (2)^+^	1	LT Heart and Circulation	8	9 (2)	4 (1)	13 (1)
Satisfaction with Care	14 (2)	3	16 (2)^+^	1	Heart and Circulation	8	9 (2)	3 (1)	14 (1)
Time to Heal Graft	13 (1)	4 (1)	16 (2)^+^	1	Fluid Amount	9 (1)	8 (1)	3 (1)	14 (1)
Anxiety About Treatment	13 (2)	4	16 (2)^+^	1	Length of Time on Ventilator	6	11 (2)	3 (1)	14 (1)
Itch	16 (1)	1 (1)	16 (1)	1 (1)	Fighting Infection	7 (1)	10 (1)	2	15 (2)^−^
LT Scar Size	17 (2)	0	16 (2)^+^	1	Nature of Exudate	3 (1)	14 (1)	2 (1)	15 (1)
LT Medication	13 (2)	4	16 (2)^+^	1	Kidney Function Serious	7	10 (2)	2 (1)	15 (1)
LT Body Temperature Regulation	12 (1)	5 (1)	16 (2)^+^	1	Liver Function	5	12 (2)	2 (1)	15 (1)
LT Wellbeing	13	4 (2)	16 (1)	1 (1)	Breathing and Lung Function	7	10 (2)	2 (1)	15 (1)
LT Sleep	15	2 (2)	16 (1)	1 (1)	Burn Smell	6 (1)	11 (1)	2	15 (2)^−^
Body Temperature	11 (1)	6 (1)	15 (2)^+^	2	Thirst	4	13 (2)	2	15 (2)^−^
Donor Site Healing	13 (2)	4	15 (2)^+^	2	ICU Neuropathy	5 (1)	12 (1)	1	16 (2)^−^
Wound Infection	12 (1)	5 (1)	15 (1)	2 (1)	Fluid Amount	5 (1)	12 (1)	1 (1)	16 (1)
Costs to the Family	12 (1)	5 (1)	15 (1)	2 (1)	Death From Burn	2	15 (2)	1	16 (2)^−^
LT Donor Site Problems	14 (2)	3	15 (2)^+^	2	Kidney Function Minor	5	12 (2)	1 (1)	16 (1)
LT Muscle Strength	11 (1)	6 (1)	15 (1)	2 (1)	Stomach and Bowel Function	6	11 (2)	1 (1)	16 (1)
LT Growth	14 (1)	3 (1)	15 (1)	2 (1)	LT Bone Strength	4	13 (2)	1	16 (2)^−^
LT Number of Outpatient Appointments	13 (1)	4 (1)	15 (2)^+^	2	Enteral Intolerance	3	14 (2)	0	17 (2)^−^
Number of Dressing Changes	13 (2)	4	14 (1)	3 (1)	Need for Transfusion	4	13 (2)	0	17 (2)^−^
LT Fitness	12 (2)	5	14 (1)	3 (1)	Death From Any Cause	1	16 (2)	0	17 (2)^−^
Donor Site Infection	10 (1)	7 (1)	13	4 (2)	LT Death From Any Cause	3	14 (2)	0	17 (2)^−^

^*^Patient votes are displayed in brackets behind the total number of votes; **LT = long term; ^+^ = decided as related to scarring; ^−^ = decided as unrelated to scarring.

#### Consensus Meeting Participation

The meeting was attended by 19 participants, including 6 patients and 13 health care professionals, originating from four countries (Table 3). Of these, 10 healthcare professionals and 1 patient had already taken part in the survey rounds. One patient and two healthcare professionals did not attend the consensus meeting. Patient panelists were adults who were burned as a child or adolescent.

**Table 3.** Demographics of consensus meeting participants

Demographics	Participants (*n* = 19)
**Gender**	
Male	6
Female	13
**Occupation**	
Doctor ([Pediatric] Burn Surgeon, Anesthetist, and Junior Doctor)	4
Allied Health Professional (Physiotherapist, Occupational Therapist, and Psychologist)	5
Burn Care Nurse/Research Nurse	0
Researcher	3
Burns Charity	1
Patient	6
Parent	0
Experience in Burn Care (Health Care Professionals)	
0–3 yr	1
4–5 yr	2
>5 yr	9
Unknown	1
Time Since Injury (Patients)	
0–3 yr	0
4–5 yr	1
6–10 yr	1
11–15 yr	0
16–20 yr	1
20+ yr	2
Unknown	1
Country of Origin	
Experts	
Australia	5
Belgium	1
The Netherlands	3
United Kingdom	4
Patients	
United Kingdom	6

#### Consensus Meeting Results

The results of the consensus meeting are displayed in Supplementary Table S2. During the meeting it was agreed 19 outcomes were related to burn scarring. The remaining 31 outcomes were regarded as not related to scarring.

The final list of outcomes agreed to be related to cutaneous burn scarring is displayed in Table 4. Outcomes are sorted in vote order, ranking from high percentage of votes to low percentage of votes.

**Table 4.** Final list of agreed outcomes

Definitely Burn Scar Related		Consider Burn Scar Related	Not Burn Scar Related	
Patient psychology	LT Anxiety (about treatment)	Donor site infection	LT Death from any cause	Amount of medication
Scar elasticity	LT patient costs	Sleep	Death from any cause	Inflammatory markers
Time to healing	LT personal relationships	Fatigue	Need for transfusion	Dressing comfort
Number of surgeries	LT return to work and school	Suicide	Enteral intolerance	Moderate complications
Adherence to treatment	Impact on family	Metabolism	LT Bone strength	Dysphagia
Anxiety about the future	Breast development	Substance abuse	Death from burn	Unplanned readmission
Understanding of treatment	Satisfaction with care	LT metabolism	ICU neuropathy	Other infection
LT* walking	Time to heal graft	Body weight	Thirst	Dignity
LT scar contractures	Anxiety about treatment	Number of appointments	Burn smell	Fluid amount
LT burn color	LT scar size	Costs to the family	Fighting infection	Multiorgan dysfunction
LT daily tasks	LT medication	Healthcare costs (long term)	Pain in the donor site	Stomach and bowel function
LT adherence to treatment	LT body temperature regulation		Heart and circulation	Multiorgan failure
LT anxiety (about the future)	Body temperature		Body weight	Fluid amount
LT appearance	Donor site healing		Heart and circulation	Kidney function minor
LT unwanted attention	LT donor site problems		Length of stay	Kidney function serious
LT understanding of treatment	LT number of outpatient appointments		Length of ICU stay	Liver function
LT use of creams and dressings	Pain all the time		Healthcare costs (short term)	Length of time on ventilator
LT number of surgeries needed	Wound infection		Serious complications	
LT number of reconstructions needed	Itch		Number of dressing changes	
LT dignity	Fitness		Sepsis	
LT hair loss	Wellbeing		Nature of exudate	
LT scar pain	Pain during treatment		Dysphonia	
LT scar texture	Muscle strength		Breathing and lung function	
LT itch	Growth		Cognitive function	

^*^LT = Long term.

## DISCUSSION

Our consensus-based findings are important in creating an understanding of the extensive impact of burn scarring and support a more holistic approach to burn scar assessment. This study identified various established and potentially important burn scar-related outcomes including anxiety, costs, fitness, metabolism, sleep, and fatigue. Outcomes that go beyond the currently most-assessed scar characteristics and psychological sequelae are considerably notable, such as the understanding of treatment and unexpected physiological issues such as body temperature, and metabolism. This array of outcomes highlight the complex impact of burn scarring on an individual.

Multiple outcomes identified in this study (Table 4) are not regularly assessed in clinical practice, nor in research. First, an important aspect of recovery after a burn injury is adaptation to the injury and restoring one’s self-image.^11,16^ Important outcomes identified in our study therein were appearance-related concerns, attracting unwanted attention, anxiety regarding the future, and dignity. Appearance concerns play an important role after a burn injury in both adult^25^ and adolescent burn patients, with a reported incidence of up to 70% in the latter.^26^ Although most become more adjusted to their new appearance over time, some still struggle to cope years after the burn injury.^27^ Jones et al identified appearance as a core outcome of interest in qualitative interviews with patients and parents of children with burn scars.^11^ Dignity during care (encompassing autonomy, being handled with respect) is an important concept, but appears to be underrepresented in burns research.^28^ During the consensus meeting, patient panelists emphasized the importance of patient support groups, stating that it helped them regain a sense of normality. Mathers et al described this “sense of normality” as one of the aspects of the “sense of self” or self-image domain in a comprehensive review of qualitative research.^16^ Mathers subsequently revealed a lack of measurement of outcomes related to this “sense of self” domain in current burns research.

Second, outcomes relating to clinical treatment were considered to be related to scarring in this study. As well as treatment strategies for scarring such as creams or reconstructions, panelists identified understanding of treatment and adherence to treatment as being important to consider (Table 4). For patients who require intensive scar management, receiving adequate information, and developing an understanding of their treatment is of importance to make informed choices about their care, and to support adherence to treatment strategies.^29^ Price et al found that providing continuity in care by the same clinicians and shared decision making regarding the treatment strategy is also important for the patient.^30^

In developed countries, healthcare costs are usually less of an issue compared to developing countries.^31,32^ Moreover, costs differ for different healthcare systems and various patient-related factors such as traveling distance. In this study, “Healthcare costs” was considered as an umbrella term for costs of burn care for the healthcare system, health insurers or to individuals. In the consensus meeting, costs for either the patient or their family were considered related to scarring, acknowledging these as important items for those who have limited financial means.

Physiological issues are beyond what is usually covered in burn scar treatment and follow-up, despite their profound impact on daily life.^33,34^ Items like fitness, hair loss, general wellbeing, body temperature, pain, muscle strength, sleep, fatigue, metabolism, LT metabolism, growth, and body weight were considered to be related to scarring by patients and clinicians in this study. Clinicians may consider these systemic outcomes to be hard to influence or beyond their remit.^35^ Nonetheless, a multidisciplinary approach and guidance throughout the rehabilitation process can aid in improving these outcomes for patients over the years.^36,37^

Scar quality characteristics such as elasticity, texture, itch, and pain were immediately selected in the first survey of this study. Scales assessing these scar characteristics such as the POSAS or VSS have been the most frequently used tools in burn research and clinical practice for many years.^9,38,39^ Since these scales mostly target visual, tactile and measurable outcomes, they are the most straightforward to use to make choices regarding treatment for burns scars. Patients have also, however, reported on other important aspects of their scars in qualitative work, such as inflammation, tightness, fragility, sensitivity, and sensation,^40–44^ which are not covered by all of the current scar scales. Similarly, patient psychological wellbeing after an injury and personal relationships were items considered to be related to scarring. These psychological and psychosocial aspects of scarring are known to impact quality of life for many years after the burn injury.^13,14,32^ Routine screening for these problems during follow-up is thus warranted. To continue optimization of contemporary treatment, it is important to involve both patient and clinical panelists’ views in determining meaningful burn scarring outcomes.

Perfect consensus on outcomes was not the aim of our study. Agreement is not realistic due to different values, world views, and ethical dilemmas concerning medical decision making.^23^

## Strengths and Limitations

A general limitation performing a Delphi study is the absence of a methodological basis. For example, there is no general threshold for defining a consensus leading to numerous variations.^22^

In this study, a threshold of 80% was chosen to reach an evident majority.

The dataset used for this research was not assembled for burn scars specifically. Instead, the COSB-i outcome long-list dataset was purposively assembled to generate consensus on a general, multidimensional Core Outcome Set for burn injuries.^19^ For the study described here, the outcomes were reviewed for relation to scarring by international health care professionals specialized in burn care, and patients not previously involved in the earlier work. The patient and healthcare professional panel was moderate in size.^22^ It consisted of professionals from various disciplines and active participation from the patient panelist was achieved. Their views even changed the perspective of experienced clinicians on some outcomes during the consensus meeting.

While we achieved participation from different countries and healthcare systems, participants were from developed countries only, and patients from the United Kingdom only, despite efforts to involve those from developing countries. This limits the generalizability of our findings. However, the outcomes identified as being relevant to burn scarring through this consensus process did not differ in terms of whether they were rated as important across country-income groups in the original COSB-i dataset.^45^ It may be useful in the future to validate whether the items selected in this study would also be selected as relevant to scarring in developing countries.

## Future Recommendations

Recently, global burns researchers were asked to further implement routine assessments of scar-related quality of life in burns aftercare.^46^ From our work we would recommend to first focus on one step before that. First, determine which outcomes should be measured (the what), before focusing on which instruments are valid or should be developed (the how). This can be achieved through research that extends the preliminary work in the current study by: 1) confirming outcomes of importance that may have been missed in the current study and existing literature; 2) prioritizing outcomes using criteria such as feasibility (ie considering burden to patients and clinicians); 3) refinement of these outcomes based on measurement approaches and feedback of outcomes in practice; 4) further explore the influence of patients participating in support groups on scar-related outcomes. Active patient participation in this decision-making process of choosing outcomes is of the utmost importance. Finally, international collaboration, prioritization and a comprehensive registry of burn outcomes, including scar-related items, would enable more patient-centered care around the globe.^47^ Consensus approaches can guide outcomes that should be considered in clinical trials and registries. However it is recognized that these approaches may not capture outcomes of importance to individual patients which should be measured alongside standardized measures derived from consensus approaches.^48^

## CONCLUSION

To represent a holistic assessment of outcomes related to cutaneous burn scarring, this Delphi process established a battery of outcomes currently included in scar quality assessment tools, and an expanded set of less frequently considered outcomes that can and should be viewed as related to scarring. Future work in this area must include the patient voice from developing countries. This is essential to identify globally applicable outcomes related to scarring.

## Supplementary Material

irad089_suppl_Supplementary_Tables

## Notes

*Authors’ contributions* EM, AD, MV, and AY contributed to the study concept and design, collected, and assembled the data. MW, EM, MV, and AY drafted the manuscript. AD, MV, AY, DE, and CM critically revised the manuscript. All authors read and approved the final manuscript.

*Ethics*. Patients were involved as a patient advisory group in concordance with the INVOLVE statement by the National Institute for Health Research (NIHR).^49^ Clinicians, burn researchers and members of burn charities were invited to take part as a professional panelist group. The methodology was discussed by the South West-Frenchay Research Ethics Committee. Additionally, the opinion from an Australian ethics committee representative was sought. Neither deemed formal approval necessary for this project. Ethical approval was therefore not required.

*Funding*. This article presents independent research funded by the National Institute for Health Research (NIHR) Advanced Research Fellowship NIHR 301362. The views expressed are those of the authors and not necessarily those of the NHS, the NIHR or the Department of Health. The study was also supported by the NIHR Biomedical Research Centre at the University Hospitals Bristol and Weston NHS Foundation Trust and the University of Bristol.
