# Supplementary material for: Working Towards Holistic Scar Assessment and Improved Shared Decision Making in Global Burn Care
Source: J Burn Care Res. 2023 Jun 13;45(1):112–9. doi: 10.1093/jbcr/irad089 (PMC10768758; doi:10.1093/jbcr/irad089)
Supplement: irad089_suppl_Supplementary_Tables [file irad089_suppl_supplementary_tables.docx]

**Supplementary table S1: List of 100 burn outcomes from round 2 of the COSB-i study.**

| **Question code** | **Full question** |
| --- | --- |
| Patient psychology | Patients' psychological wellbeing following injury. For example: Post-traumatic Stress Disorder (PTSD), depression, mental state, need for psychological support. |
| Impact on family | Psychological impact on the patient's parents, partner, relatives or friends. For example: trauma from witnessing an accident, distress, depression, anxiety, need for psychological support. |
| Suicide | Suicide rate. For example: number of patients who die by suicide following burn injury. |
| Breast development | Breast development. For example: some female patients who experience a burn or scald to the chest area may require further treatment during breast development to release scars. |
| Substance abuse | Substance abuse or addiction. For example: abuse or addiction to alcohol or prescribed painkillers following treatment. |
| Dysphagia | Difficulty swallowing (Dysphagia). For example: some patients experience difficulty swallowing due to injury to their throat or because they have had a tube to help them breathe while they were in intensive care. |
| Dysphonia | Difficulty speaking (Dysphonia). For example: Some patients have difficulty speaking due to injury to their throat or because they have had a tube to help them breathe while they were in intensive care. |
| Fatigue | Tiredness (fatigue) For example: some patients experience persistent tiredness for a long time after burn injury. |
| ICU Neuropathy | Intensive care neuropathy. For example: some patients experience long term physical weakness after staying in an intensive care unit which may or may not improve overtime. |
| Enteral intolerance | Enteral feeding intolerance. For example: some patients require a tube to give them food so that they receive adequate nutrition. Enteral feeding intolerance is a lack of ability to absorb this food. |
| Unplanned readmission | Unplanned readmission to hospital. For example: needing to be readmitted to hospital unexpectedly. This might be due to infection or break down of the wound. |
| Satisfaction with care | Satisfaction with care. For example: the patient or their family's satisfaction with the care they have received. |
| Scar elasticity | Burn scar elasticity. For example: how well the scar stretches when the person moves, how tight their skin feels. |
| Fighting infection | How well a patient with a burn is able to fight infection. For example: a burn can affect the body's ability to fight infection. Medical tests like white cell count can show how well the body can fight infection. |
| Fluid amount | The amount of fluid (exudate) coming from the burn wound. For example: whether and how much the burn leaks fluid. |
| Nature of exudate | The nature of the fluid coming from the burn wound. For example: what colour is the fluid coming from the burn wound, and has it changed? |
| Need for transfusion | The need for blood transfusions during treatment for a burn. For example: does a patient need to be given blood or blood products during their treatment? This sometimes happens if the burn is large or the patient needs an operation for a skin graft. |
| Body temperature | The difficulty patients have with body temperature. For example: A burn can affect the way the body handles temperature by increasing a patient's sensitivity to heat or by being unable to sweat. |
| Time to healing | How quickly a patient's burn wounds heal. For example: how many days or weeks does it take for the burn to heal completely. |
| Time to heal graft | How quickly a patient's burn wound heals after receiving a skin graft. A skin graft is when healthy skin is taken from another part of the body and placed over the burn wound to help it heal. For example: how well a burn that has needed a skin graft heals. |
| Donor site healing | How quickly the donor site heals in patients who have had a skin graft. Donor site: is the place from which healthy skin is taken for a skin graft- usually the top of the thigh. For example: how soon does the site where the skin graft has come from heal? |
| Donor site infection | Whether the donor site becomes infected. Donor site: is the place from which healthy skin is taken for a skin graft-usually the top of the thigh. For example: whether the area of the body that the skin is taken from for a skin graft becomes infected. |
| Wound infection | Whether a burn wound becomes infected. For example: burn wounds may become infected because they have lost the outer layer of the skin. This will require treatment such as wound cleaning and/or antibiotics. |
| Other infection | Whether a patient has an infection elsewhere in the body, other than in the burn wound. For example: chest infection, urine infection. |
| Sepsis | Whether a burn results in bloodstream infection (sepsis). For example: severe infection in the blood with a risk to life. |
| Moderate complications | Whether patients experience moderate complications relating to the burn or its treatment, which will get better with treatment but may affect the patient's length of stay in hospital. For example: allergy to medication or bleeding under their skin graft. |
| Serious complications | Whether patients experience serious complications relating to the burn or its treatment, which could result in death, or require considerable treatment, and may considerably extend the hospital stay. For example: blood clots in the lungs or legs from lying in bed. |
| Death from burn | Death due directly to the burn injury soon after the patient is injured. For example: death due to 'burn shock' or due to a burn wound infection or sepsis. |
| Death from any cause | Death of a patient from any cause soon after the patient is injured. For example: death from a heart attack. |
| Metabolism | The effect of the burn on how well the body uses energy (hypermetabolic response). For example: the body may have a very high use of energy when trying to heal wounds so that muscles become weak and there is weight loss. |
| Heart and circulation | The effect of the burn on a patient's heart and blood circulation function. Large burns can affect a patient's heart function. For example: low blood pressure requiring intensive care and drugs, which could be life threatening, or high blood pressure. |
| Kidney function minor | Effect of the burn on a patient's kidney function that does not require dialysis. Burns can affect the functioning of a patient's kidneys. The kidneys filter waste products out of your blood. Dialysis is a machine that does the work a patient's kidneys normally do. For example: minor effects of the burn on the working of a patient's kidneys that requires drugs or more fluid. |
| Kidney function serious | Kidney failure caused by the burn that requires dialysis. For example: serious effect of the burn on a patient's kidneys that means a machine is needed that does the work of the kidneys (dialysis), which may or may not be permanent. |
| Liver function | The effect the burn has on the patient's liver function. For example: rarely a burn can affect the patient's liver because of changes in the patient’s blood pressure, or because of infection. This would be serious and affect a patient's ability to clot their blood normally, increase their time in hospital, and if very serious may be a risk to life. |
| Breathing and lung function | Whether the patient with a burn has any difficulty with breathing or lung function. For example: a small number of patients with burns have problems with their breathing or lungs due to inhaling smoke, or because the burn or fluid given to treat the burn can affect the lungs. This might mean that a patient needs to be helped to breathe with a ventilator or use oxygen. |
| Stomach and bowel function | The effect of a burn on the function of a patient's stomach or bowel. For example: diarrhoea, constipation, sickness, nausea, inability to keep food down. |
| Multiorgan dysfunction | Whether the burn causes several body organs to stop working well at the same time (multi-organ dysfunction). After a burn a patient can develop problems with several organs at once (called multi-organ dysfunction), but with treatment the organs can recover. For example: poor kidney function and poor liver function at the same time that is likely to get better following treatment. |
| Multiorgan failure | Whether the burn causes several of the patient's organs to fail (not work at all) at the same time (multi-organ failure). Rarely, a patient with a burn can develop organ failure in several organs at once (called multi-organ failure). For example: kidney failure alongside liver failure, where it is unlikely to get better, or will need long term care. |
| Fluid amount | The amount of fluid given to a patient, either into a patient's vein (through a 'drip'), or as a drink. When a patient loses skin due to a burn they will lose liquid through this area, and if it is a large area the fluid may need to be replaced. For example: how much extra fluid through a drip does a patient need to have to ensure their organs work well. |
| Length of stay | The length of time a patient stays in hospital after a burn injury. For example: the number of days or weeks after the injury that a patient needs to stay in hospital. |
| Length of ICU stay | The length of time a patient stays in an intensive care unit after a burn injury. For example: the number of days or weeks after the injury that a patient has to receive intensive care. |
| Length of time on ventilator | The length of time a patient uses a breathing machine after a burn injury. For example: the amount of time a patient needs to be on a 'ventilator'. |
| Inflammatory markers | Medical tests to find out how well the body is handling the stress of the burn injury (inflammatory markers). For example: blood or urine tests to find out how well the body is coping with the stress placed on the body after burn injury. |
| Body weight | Whether a patient can maintain their body weight after a burn injury. For example: burn injuries can affect a patient's ability to eat normally and absorb their food. This can result in weight loss, or not being able to keep to a normal weight. |
| Healthcare costs | The costs of burn treatment for the healthcare systems such as the NHS (UK). For example: how much the treatment for the burn costs the NHS/hospital through paying for staff, dressings, medications and equipment. |
| Number of dressing changes | The amount of dressing changes or cream applications needed to treat a burn. For example: how many times a patient needs to have their dressing changed, for how long and how frequently they have to apply creams. |
| Number of appointments | The number of outpatient appointments a patient needs to attend. For example: how many times a patient needs to come to the hospital for follow-up appointments or dressing changes. |
| Number of surgeries | The number of surgical treatments/operations a patient needs. For example: the number of times they need to have an operation or receive a treatment where they are put to sleep. |
| Burn smell | The smell of the burn wound. For example: does the burn wound smell unpleasant. |
| Amount of medication | How much medication a patient needs to treat a burn injury. For example: how many painkillers are needed or whether a patient needs medication for blood pressure. |
| Adherence to treatment | How well a patient sticks to their planned treatment. For example: do patients take medication, do they attend appointments and have blood tests as directed by their medical team? |
| Anxiety about treatment | The anxiety a patient experiences about their medical treatment. For example: worry about the pain of operations, dressing changes, blood tests. |
| Dressing comfort | How comfortable wound dressings are for a patient. For example: do the dressings fall off, do they stop the patient moving normally, are they itchy? |
| Dignity | The dignity of the patient during and after treatment. For example: does the patient feel respected, are they given privacy? |
| Itch | Itch in the burn wound during healing of the burn. |
| Pain all the time | Pain in the burn wound when a treatment is not taking place. For example: pain all the time, pain at night. |
| Pain during treatment | The amount of pain caused by medical procedures for a patient with a burn. For example: pain when having dressing changes, blood tests. |
| Pain in the donor site | Pain in the donor site. Donor site: is the place from which healthy skin is taken for a skin graft- usually the top of the thigh. For example: how sore is the area where the skin is taken from for a graft. |
| Anxiety about the future | A patient's anxiety about the future. For example: worries about appearance, worries about working or school, worries about relationships. |
| Costs to the family | The effect being treated for a burn has on a patient or their family in terms of money. For example: lost salary for a patient or carer, costs of travel to appointments for patients and their family, buying food at the hospital, costs of painkillers and prescriptions. |
| Thirst | The effect of the burn and treatment on a patient's thirst. For example: feeling so thirsty that a drink of water does not make it better. |
| Understanding of treatment | How much a patient understands the treatment they receive for a burn injury. For example: whether the patient understands the need for surgery, how long scar treatment will take. |
| LT Bone strength | The effect of a burn on the strength of a patient's bones after healing. For example: a burn injury can affect a patient's bones. This might include a change in bone density, brittle bones with an increased risk of broken bones (osteoporosis). |
| LT Healthcare costs | The costs of burn treatment for the healthcare system such as the NHS (UK). For example: how much treatment for the burn and scar costs the NHS/hospital through paying for staff, dressings and medications and equipment. |
| LT Death from any cause | Death of a patient from any cause. For example: death from a stroke or a heart attack after the burn has healed. |
| LT Metabolism | The effect of the burn on how well the body uses energy. For example: a patient's body may need to use a lot of energy during healing so that muscles become weak and there is weight loss. |
| LT Heart and circulation | The effect of the burn on a patient's heart and blood circulation function. For example: a burn injury can affect a patient's heart functioning or circulation. A long-term effect after healing might include the need for bloodpressure treatment. |
| LT Donor site problems | Whether there are problems with the skin graft donor site after healing. Donor site: is the place from which healthy skin is taken for a skin graft- usually the top of the thigh. For example: pain, colour change of the donor site after it has healed. |
| LT Walking | How much the burn affects a patient's ability to walk. For example, walking speed, not shuffling. |
| LT Scar Contractures | The effect of the burn scar on a patient's ability to move joints (contractures). For example, inability to straighten arm, difficulty moving fingers normally, limited range of motion of joints. |
| LT Fitness | The effect of the burn (and treatment) on a patient's fitness. For example: ability to walk as far as normal, being able to do exercise, lack of energy, breathless on walking or stairs. |
| LT Muscle strength | The effect of the burn on the strength of a patient's muscles. For example: poor muscle strength, difficulty with carrying children or shopping. |
| LT Body weight | Whether a patient can maintain their body weight after a burn injury, after healing. For example: weight loss, notable to keep to a normal weight. |
| LT Growth | The effect a burn has on a child's growth. A burn can rarely affect a child's growth. For example: not achieving potential height, slowing of growth. |
| LT Burn colour | The difference in colour of a burn scar compared to normal skin. For example, whether a burn scar is very red, or loss of colour in a scar. |
| LT Scar size | The size of a burn scar. For example, the size of the final scar, not the original burn. |
| LT Medication | How much medication a patient needs to manage the burn scar and other symptoms after the injury. For example, whether a patient requires medication for a long time after the injury, how many painkillers are needed. |
| LT Daily tasks | A patient's ability to carry out normal daily tasks. For example: dressing, washing, making food or drinks. |
| LT Adherence to treatment | How well a patient sticks to their planned treatment. For example, do patients take medication, do they attend appointments and have blood tests. |
| LT Anxiety (about treatment) | The anxiety patients experience about their medical treatment. For example: worry about the pain of operations needed for treatment of scars. |
| LT Appearance | Patients' appearance after a burn injury. For example, appearance of the scar, facial appearance, body image. |
| LT Body temperature regulation | The difficulty patients have with body temperature management after a burn. For example: sensitivity to heat, being unable to sweat. |
| LT Unwanted attention | How much the burn results in a patient experiencing unwanted attention. For example: people looking, judgement by others, name calling. |
| LT Understanding of treatment | How much a patient understands the treatment they are receiving for their burn injury. For example: whether a patient understands the need for surgery, scar treatments. |
| LT Use of creams and dressings | The amount of cream applications, or amount of time wearing pressure garments needed to treat the scar. For example, for how long and how frequently a patient has to apply creams or use pressure garments for their scars. |
| LT Number of outpatient appointments | The number of outpatient appointments a patient needs to attend. For example: how many times a patient needs to come to the hospital for follow-up appointments. |
| LT Number of surgeries needed | The number of surgical treatments or operations a patient needs. For example, the number of times they need to have an operation or receive a treatment to help with scarring, where they are put to sleep. |
| LT Number of reconstructions needed | How much surgery is needed to treat a patient's scars. For example, how many times their scar may need surgery. |
| LT Dignity | The dignity of a patient during scar treatment. For example: does the patient feel respected, are they given privacy. |
| LT Hair loss | The loss of a patient's hair due to the burn injury. |
| LT Scar pain | The amount of pain caused by a burn scar. |
| LT Scar texture | The texture or feel of a burn scar. For example, whether a scar is rough, lumpy or tight. |
| LT Itch | Whether the burn causes a patient to have problems with itch after healing. For example, some patients have itchy scars that can affect their ability to sleep, and take part in daily activities. |
| LT Anxiety (about the future) | A patient's anxiety about the future. For example, worries about appearance, worries about working or school, worries about relationships. |
| LT Patient costs | The effect that being treated for a burn has on a patient or their family in terms of money. For example, lost salary for a patient or their carer, costs of travel to appointment and parking for a patient or their family, buying food at the hospital, costs of painkillers and prescriptions. |
| LT Cognitive Function | The effect of the burn on a patient's ability to think and remember clearly. For example: memory, concentration. |
| LT Wellbeing | The effect a burn has on general well-being. For example: don't feel right, general illness. |
| LT Sleep | How much a burn affects the amount and quality of sleep a patient gets. |
| LT Personal relationships | How the burn injury and treatment affect a patient's personal relationships. For example: relationships with parents, boyfriend, girlfriend, partner, children, friends, brothers and sisters. |
| LT Return to work and school | How long a burn prevents a patient from returning to work or a child or young person returning to school, University or College. |

**Supplementary table S2: Responses in the consensus meeting.**

| **Domain** | **Outcomes** | **Voted: relates**  **to scarring (%)** | **Voted: does not**  **relate to scarring (%)** | **Voted: don’t know (%)** |
| --- | --- | --- | --- | --- |
| Psycho(social) | Suicide  Substance abuse  Dignity | 72  67  17 | 17  17  78 | 11  17  6 |
| Burn wound related | Fluid amount  Nature of exudate  Donor site infection  Wound infection  Itch | 17  28  78  94  94 | 72  61  11  0  0 | 11  11  11  6  6 |
| Complications | Other infection  Sepsis  Moderate complications  Serious complications | 18  29  24  35 | 76  71  76  53 | 6  0  0  12 |
| Dysphagia/dysphonia | Dysphagia  Dysphonia | 22  28 | 78  72 | 0  0 |
| Short term systemic effects | Fatigue  Metabolism  Body weight | 74  68  63 | 11  16  21 | 16  16  16 |
| Organ dysfunction or failure | Heart and circulation  Kidney function minor  Kidney function serious  Liver function  Breathing and lung function  Stomach and bowel function  Multiorgan dysfunction  Multiorgan failure | 56  0  0  0  28  11  17  11 | 39  94  94  94  67  83  83  83 | 6  6  6  6  6  6  0  6 |
| Pain and discomfort | Pain all the time  Pain during treatment  Pain in the donor site  Dressing comfort | 100  84  58  26 | 0  16  32  68 | 0  0  11  5 |
| Long term (systemic) effects | LT* Metabolism  LT Heart and circulation  LT Fitness  LT Muscle strength  LT Body weight  LT Growth  LT Cognitive function  LT Wellbeing  LT Sleep | 67  39  94  83  50  83  28  94  78 | 33  50  6  11  39  17  67  0  17 | 0  11  0  6  11  0  6  6  6 |
| Hospital stay | Unplanned readmission  Length of stay  Length of ICU** stay | 22  39  39 | 72  61  61 | 6  0  0 |
| Treatment | Length of time on ventilator  Number of dressing changes  Number of appointments  Amount of medication  Fluid amount  Inflammatory markers | 0  33  61  28  11  28 | 100  67  39  67  89  61 | 0  0  0  6  0  11 |
| Costs | Healthcare costs (short term)  Costs to the family  LT Healthcare costs | 39  61  61 | 56  33  39 | 6  6  0 |

*LT = long term

**ICU = Intensive Care Unit
